# Supplementary material for: Evaluating the Effect of a Novel Digital Ostomy Device on Leakage Incidents, Quality of Life, Mental Well-Being, and Patient Self-Care: An Interventional, Multicentre Clinical Trial
Source: J Clin Med. 2024 Sep 24;13(19):5673. doi: 10.3390/jcm13195673 (PMC11477101; doi:10.3390/jcm13195673)

Supplementary Table S1. Serious adverse events.

#1	- Subarachnoid hemorrhage and encephalitis	Not related
#2	- Low neutrophil count	Not related
#3	- Bleeding stoma, potentially from food poisoning<br>- Dehydration, potentially from food poisoning<br>- Diarrhea, potentially from food poisoning<br>- Vomiting, potentially from food poisoning	Not related<br>Not related<br>Not related<br>Not related
#4	- High output of black stoma contents	Not related
#5	- Abdominal pain<br>- Intestinal blockage<br>- Intestinal infection	Not related<br>Not related<br>Not related

Supplementary Table S2. Non-serious adverse events.

#1	- Skin redness under arm of sensor layer, area red and itchy	Causal relationship
#2	- Operation for injury on finger<br>- Skin redness 6 cm from stoma on the abdomen	Not related<br>Causal relationship
#3	- Chest pain - diagnosed with Pulmonary Embolism<br>- Covid 19 Positive	Not related<br>Not related
#4	- Abdominal pain and vomiting<br>- Generally unwell, lethargy, changes in urine, dehydration, output very liquid	Not related<br>Unlikely related
#5	- Covid 19 Positive<br>- Headache<br>- Temperature	Not related<br>Not related<br>Not related
#6	- Covid 19 Positive<br>- Parastomal Hernia	Not related<br>Unlikely related
#7	- Soreness and bleeding around the stoma, on the hole. Also had this problem previously<br>- Red and itching skin beneath tail of Test Product	Unlikely related<br>Possibly related
#8	- Small sore near stoma. It has been coming and going since stoma formation.	Unlikely related
#9	- Red raw skin reported, painful and burning sensation, redness area reported as the size of stoma bag	Probably related
#10	- Skin rash caused by the adhesive from the baseplate	Possibly related
#11	- Skin irritation caused by the adhesive agent used on the baseplate	Causal relationship
#12	- Feelings of urgency to have bowels open rectally<br>- Fatigue<br>- Itchy peristomal skin small area less than $10cm^2$<br>- Red itchy peristomal skin (whole circumference)<br>- Red peristomal skin, small area less than $10cm^2$	Unlikely related<br>Not related<br>Probably related<br>Probably related<br>Probably related
#13	- Red, itchy, dry under Test Product 'tail'	Probably related
#14	- Dental extraction of tooth<br>- Headache<br>- Sore Throat<br>- Tooth pain<br>- Fatigue	Not related<br>Not related<br>Not related<br>Not related<br>Not related
#15	- Itchy skin under transmitter	Possibly related
#16	- Both cheeks on face itchy, red and dry<br>- Itchy skin underneath the 'tail' of Test Product	Not related<br>Causal relationship
#17	- Skin redness is directly under the 'tail' of Test Product	Probably related
#18	- Coryzal symptoms<br>- Diarrhea<br>- Fatigue<br>- Feels chilled/shivery<br>- Headache<br>- Headache<br>- Sore peristomal area under base plate	Not related<br>Unlikely related<br>Not related<br>Not related<br>Not related<br>Not related<br>Probably related
#19	- Patient reports some discharge from the back-passage	Not related
#20	- Diagnostic Sigmoidoscopy<br>- Patient reports to having some discharge coming through the back passage	Not related<br>Not related

#21	- Feeling of numbness in head when feeling panicky<br>- Headache<br>- Peristomal ulcer	Not related<br>Not related<br>Possibly related
#22	- Small area of redness around perimeter of stoma	Probably related
#23	- Arthritic pain in right wrist<br>- Headache<br>- Tension headache<br>- Redness around the edge of baseplate and stoma	Not related<br>Not related<br>Not related<br>Probably related
#24	- Back Pain<br>- Hay fever<br>- Lower left back pain<br>- Redness at the underside of stoma crescent shaped	Not related<br>Not related<br>Not related<br>Probably related
#25	- Back pain	Not related
#26	- Cold sore<br>- Dehydration<br>- Exhaustion<br>- Flare up of eczema on right hand<br>- Increase of rectal discharge (blood and mucous)<br>- Inflammation of right wrist joint<br>- Injury to stoma<br>- Trauma injury to right foot<br>- Watery output from stoma<br>- Skin redness around the periphery of baseplate	Not related<br>Not related<br>Not related<br>Not related<br>Unlikely related<br>Not related<br>Not related<br>Not related<br>Not related<br>Possibly related
#27	- Headache<br>- Headache	Not related<br>Not related
#28	- Covid-19<br>- Feeling generally unwell<br>- Joint Pain in ankles and knees<br>- Joint Stiffness in ankles and knees<br>- Watery output from stoma<br>- Stoma bleeding<br>- Stoma increased redness	Not related<br>Not related<br>Not related<br>Not related<br>Unlikely related<br>Possibly related<br>Probably related
#29	- Flexi sigmoidoscopy<br>- Pre-planned defaecating proctogram<br>- Stoma shrunk due to dehydration	Not related<br>Not related<br>Not related

Supplementary Figure S1. Impact of time since hospital discharge on baseline values of the *Emotional impact* domain. Linear regression used to test if slope equal to zero.

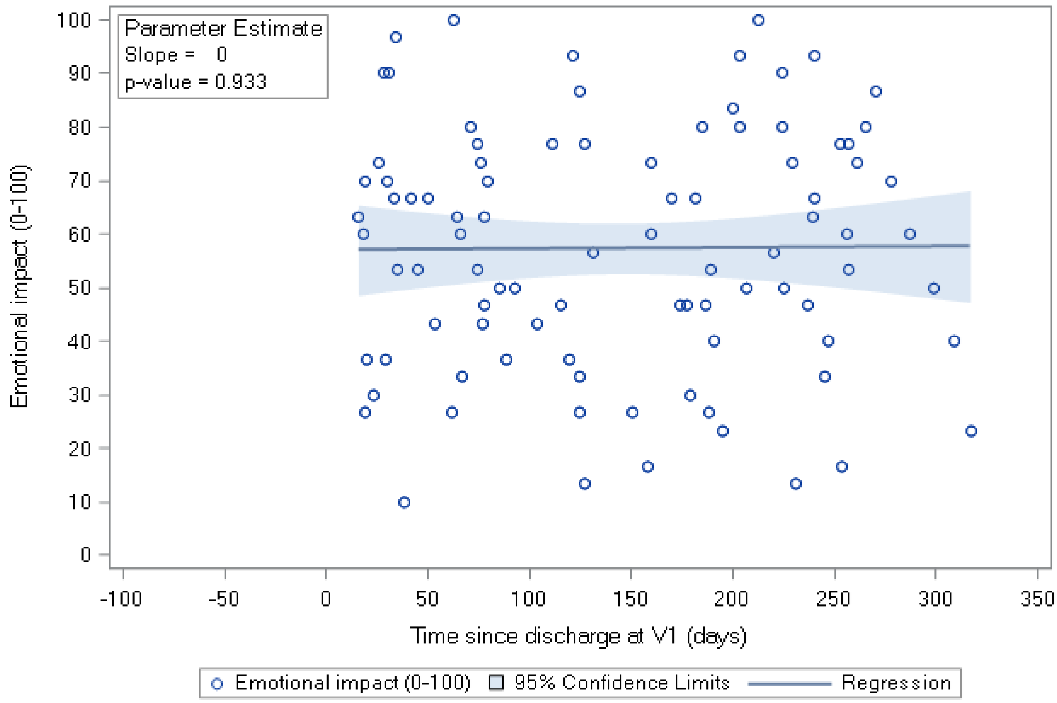

Supplement: Supplementary file 1 [file jcm-13-05673-s001.zip › jcm-3191439-supplementary.pdf]
